# Evaluation of a New Multiparameter Brain Probe for Simultaneous Measurement of Brain Tissue Oxygenation, Cerebral Blood Flow, Intracranial Pressure, and Brain Temperature in a Porcine Model

**DOI:** 10.1007/s12028-018-0541-9

**Published:** 2018-06-11

**Authors:** Marius M. Mader, Anna Leidorf, Andreas Hecker, Axel Heimann, Petra S. M. Mayr, Oliver Kempski, Beat Alessandri, Gabriele Wöbker

**Affiliations:** 1 Institute for Neurosurgical Pathophysiology, University Medical Center of the Johannes Gutenberg-University, Langenbeckstr. 1, 55131 Mainz, Germany; 2 Department of Neurosurgery, University Medical Center Hamburg-Eppendorf, Hamburg, Germany; 3 HELIOS Universitätsklinikum Wuppertal, University Witten/Herdecke, 42283 Wuppertal, Germany

**Keywords:** Neurophysiological monitoring, Swine, Brain injuries, Intracranial pressure, Oxygen, Laser Doppler flowmetry

## Abstract

**Background:**

A novel multiparameter brain sensor (MPBS) allows the simultaneous measurement of brain tissue oxygenation (ptiO_2_), cerebral blood flow (CBF), intracranial pressure (ICP), and brain temperature with a single catheter. This laboratory investigation evaluates the MPBS in an animal model in relation to established reference probes.

**Methods:**

The study group consisted of 17 juvenile male pigs. Four MPBS and four reference probes were implanted per pig and compared simultaneously. The measured parameters were challenged by standardized provocations such as hyperoxia, dobutamine, and norepinephrine application, hypercapnia and hypoxia in combination with and without a controlled cortical impact (CCI) injury. Mean values over 2 min were collected for predefined time points and were analyzed using Bland–Altman plots.

**Results:**

The protocol was successfully conducted in 15 pigs of which seven received CCI. ICP and ptiO_2_ were significantly influenced by the provocations. Subtraction of MPBS from reference values revealed a mean difference (limits of agreement) of 3.7 (− 20.5 to 27.9) mm Hg, − 2.9 (− 7.9 to 2.1) mm Hg, and 5.1 (− 134.7 to 145.0) % for ptiO_2_, ICP, and relative CBF, respectively.

**Conclusions:**

The MPBS is a promising measurement tool for multiparameter neuromonitoring. The conducted study demonstrates the in vivo functionality of the probe. Comparison with standard probes revealed a deviation which is mostly analogous to other multiparameter devices. However, further evaluation of the device is necessary before it can reliably be used for clinical decision making.

**Electronic supplementary material:**

The online version of this article (10.1007/s12028-018-0541-9) contains supplementary material, which is available to authorized users.

## Introduction

Intensive care treatment of patients with traumatic brain injury (TBI) aims at maintaining an adequate brain perfusion and oxygenation to prevent secondary brain damage [1]. A continuous neuromonitoring via intraparenchymal sensor allows assessment of pathological changes, prediction of outcome, and guidance throughout the treatment. Key parameters are particularly intracranial pressure (ICP) and brain tissue oxygen tension (ptiO_2_) since several studies could show a benefit if monitored [2–9].

Most probes in clinical use measure a single parameter only. Consequently, multiparametric monitoring usually requires simultaneous implantation of several probes with an additional risk of complications like bleeding or infection. The development of multiparameter probes addresses this issue. Until recently, the Neurovent-PTO monitor (Raumedic) has been the only probe able to measure both ICP and ptiO_2_ in a single catheter in combination with brain temperature measurement [10, 11]. A new Multiparameter Brainsensor (MPBS, Oxford Optronix Ltd., Abingdon, UK, in collaboration with Millar Instruments, Houston, TX, USA) adds laser Doppler flow (LDF) analysis of cerebral blood flow (CBF) to this lineup. First in vitro and in vivo investigations of the ptiO_2_ sensor already demonstrated proper functioning [12, 13].

The main goal of this study was to evaluate the functionality of the MPBS in comparison with well-established reference probes in a pig model under control and in order to increase heterogenicity of collected data under post-traumatic conditions. An additional goal was to confirm a previously described testing protocol that challenges different parameters in order to standardize the evaluation process for new sensors [13].

## Methods

All experiments were approved by the ethical committee for Animal Use and Care and performed according to national guidelines for animal experiments. Seventeen juvenile male pigs at the age of 3–4 months (German breed 29–32 kg) were used. Anesthesia was induced via intramuscular injection of ketamine (15 mg/kg) and azaperone (3 mg/kg) followed by intravenous administration of 10 ml thiopental (25 mg/ml; Trapanal, Nycomed). Continuous intravenous application of thiopental (10–15 mg/kg bw/h) and piritramide (0.2–0.3 mg/kg bw/h; Dipidolor, Janssen-Cilag Pharmaceuticals Inc.) maintained sedation. All animals were intubated (Lo-Contour Murphy, Mallinckrodt; i.d/o.d. 6.0/5.5 mm) and mechanically ventilated with a fraction of inspired oxygen (FiO_2_) of 0.27 (900B; Siemens-Elema AB). Body temperature was kept at a physiological level (Homeothermic Blanket Systems, Harvard Apparatus). Cannulation of femoral artery, femoral vein, and jugular vein permitted monitoring of blood pressure, blood gases, and hemodynamic parameters via PiCCO plus (PULSION Medical Systems AG, München, Germany).

**Fig. 1:**
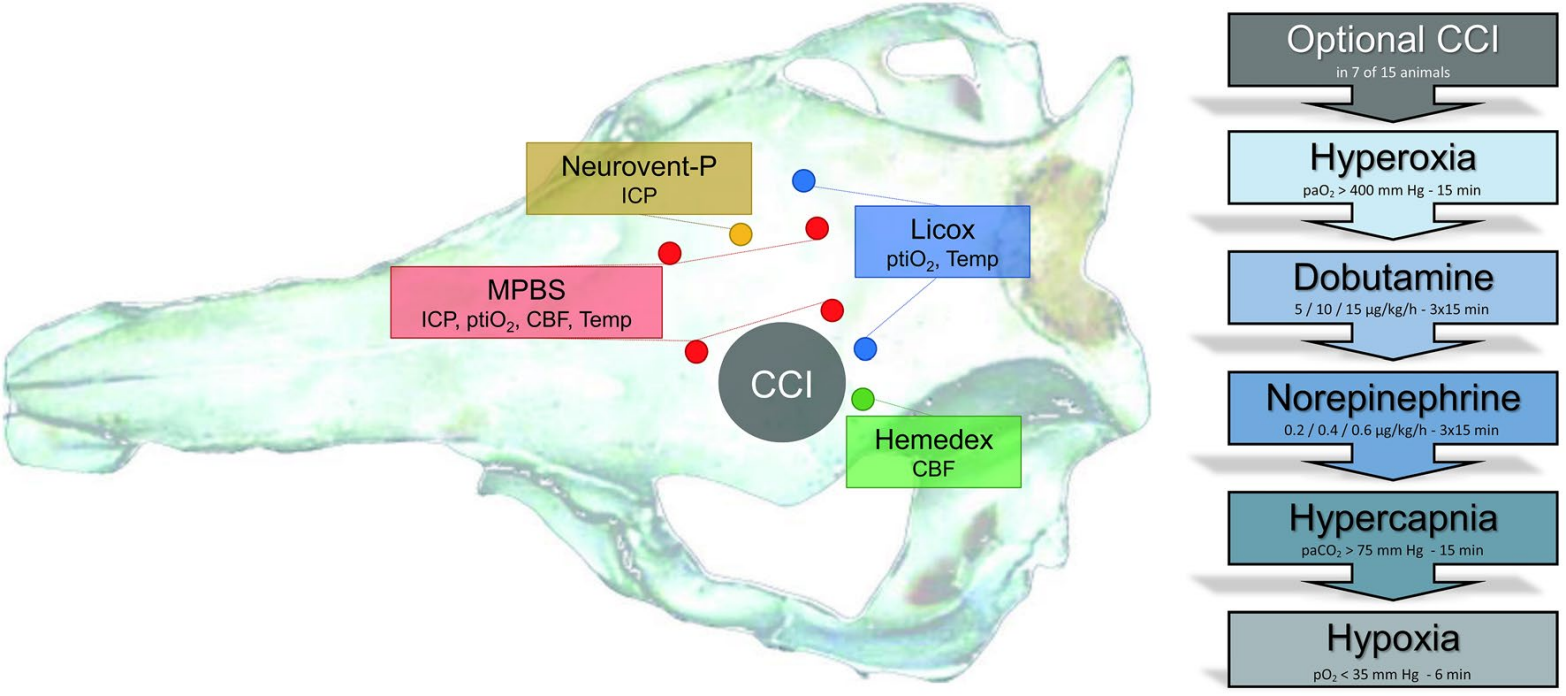
Overview of probe positioning and the protocol. The diagram demonstrates schematically the positions of the different brain probes and location of the optional craniectomy for CCI (modified after Timaru-Kast et al. [35]). The right-sided flowchart gives an overview of the protocol

**Fig. 2:**
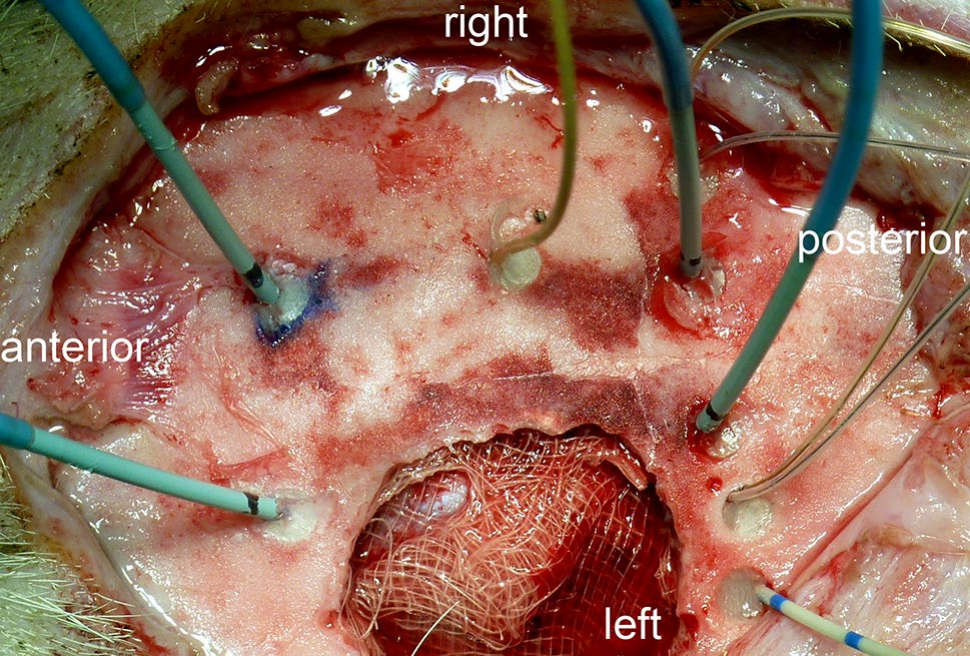
Intraoperative photography after probe positioning and craniectomy. The picture shows the exposed skull with a left parietotemporal craniectomy of an animal of the CCI group from a left superior oblique angle. MPBS and reference probes are implanted through eight burr holes

The head was fixed in a stereotactic frame, and the skull was exposed. Thereafter, four burr holes per hemisphere were drilled through the parietal bone. Nine animals additionally received a left parietotemporal craniectomy 1 cm lateral to the sagittal suture with a diameter of 3 cm. A controlled cortical impact (CCI) device was brought in position above the intact dura for later trauma induction. The dura underneath the burr holes was opened with a needle. Four MPBS and four reference probes were implanted per pig to a depth of 15 mm and fixated with bone wax (Figs. 1, 2).

**Fig. 3:**
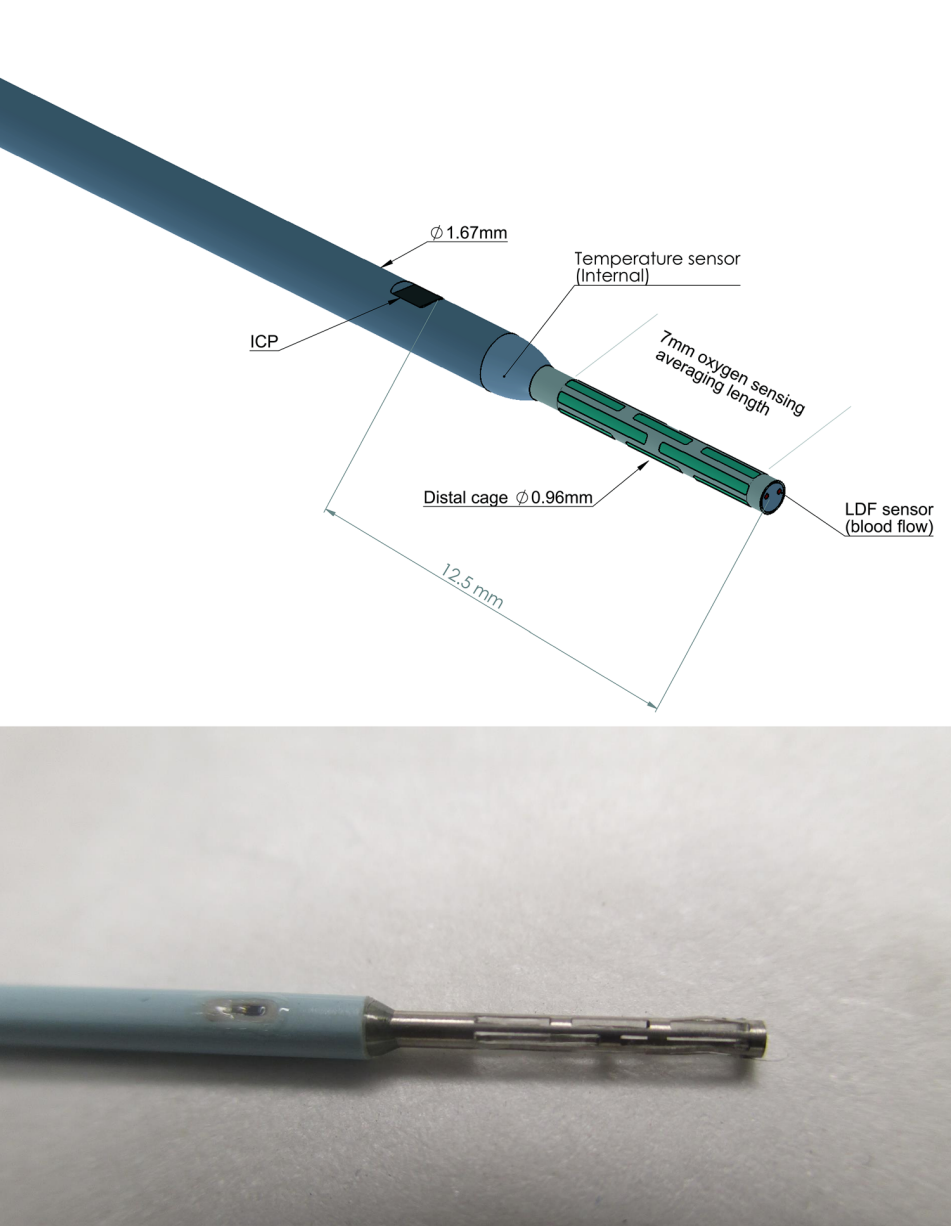
Schematic illustration and photography of the MPBS. The computer-aided design drawing demonstrates the assembly of the different sensors as well as the dimension of the MPBS. The picture shows the tip of the MPBS with the ICP sensor on the left and distal steel cage on the right

The MPBS consists of four units measuring oxygen tension, temperature, pressure, and blood flow, which are arranged along a rigid steel shaft (length 14.5 mm; diameter max. 1.67 mm [5F], tip 0.96 mm) (Fig. 3).

PtiO_2_ measurement is based on oxygen quenching. A fluorophore within a silicone matrix absorbs light pulsed through a fiber-optic light guide. Since resulting fluorescence lifetime is inversely proportional to the concentration of dissolved oxygen, oxygen tension can be calculated. This process is temperature dependent, but is corrected by the integrated thermocouple, which allows the measurement of brain temperature. No oxygen is consumed. The sensor was already precalibrated by the manufacturer. Two Licox probes (Integra Neuroscience) per pig were used as reference probes for ptiO_2_ and temperature. According to manufacturer information, the Licox oxygen probe and temperature probe have a diameter at tip of 0.6 and 0.8 mm, respectively. The Licox ptiO_2_ sensor is a Clark-type electrode: The reduction of oxygen results in a current proportional to the oxygen tension, and a small amount of oxygen is consumed.

The MPBS contains a Millar solid-state Micro-Electro-Mechanical Systems sensor for pressure measurement. A piezoresistive bridge assembly transduces pressure into a gaugeable current. The Neurovent ICP reference probe (Neurovent-P, Raumedic; dimension: 5F) is based on the same technology. One Neurovent probe was implanted per animal.

The MPBS measures CBF via LDF. This sensor consists of two optical light guides, one for laser emission and one for collection of light. Laser light is scattered by immobile tissue as well as moving particles such as erythrocytes. The difference in the reflected wavelengths results in a laser Doppler shift which is detected and calculated as a relative arbitrary unit blood perfusion unit. One thermal diffusion reference probe (Bowman Perfusion Monitor, Hemedex) was used per pig, which calculates CBF by determination of the power dissipated by a heated thermistor and has a diameter of 1 mm [14].

All ICP sensors were calibrated before implantation. Additionally, Licox probes were calibrated and all ptiO_2_ sensors were in vitro tested in oxygenated water and deoxygenated solution (0.26 g Borax (sodium tetraborate, Nr. 6306, Fa. Merck), 1.63 g sodium sulfite (Nr. 6657, Fa. Merck), 1000 ml dest. water) either before implantation or after explantation.

After sensor implantation (15 mm insertion depth) and an equilibration period of 60–120 min, the study protocol consisting of optional CCI as well as pharmaceutical and respiratory manipulations was started (Fig. 1). The protocol was based on a previously published methodical groundwork [13]. After surveying baseline values, the nine craniectomized pigs underwent CCI. Intention for CCI was to induce an additional aspect of interindividual heterogeneity including alterations in cerebrovascular autoregulation. Trauma parameters were a velocity of 3.5 m/s, depth of 10 mm, and duration of 200 ms. Afterward, the craniotomy was closed with an alginate plastic. Effects of the trauma were observed over a period of 30 min before further provocations began. Respiratory challenges included hyperoxygenation (paO_2_ > 400 mm Hg; 15 min), hypercapnia via apnoeic oxygenation for 15 min (paCO_2_ > 75 mm Hg) and hypoxia by ventilating with an air/N_2_ mixture for approximately 6 min (pO_2_ < 35 mm Hg). Pharmaceutical manipulations were comprised of an administration of dobutamine (5/10/15 µg/kg/h; 3 × 15 min; Carinopharm GmbH) and norepinephrine (0.2/0.4/0.6 µg/kg/h; 3 × 15 min; Arterenol, Sanofi-Aventis). There was an at least 15-min recovery period between the respective challenges.

Data were recorded at 1 Hz using LabChart Software (ADInstruments) and analyzed with Sigmaplot (Systat Ldt.). Mean values of 2 min were calculated before each challenge and every 5 min (every 2 min during hypoxia) during the challenges. The averaged values were then analyzed in Bland–Altman plots [15, 16]. Line plots were created comparing mean values of MPBS and reference probes. Applying an α-level of 0.05, Mann–Whitney rank-sum test or *t* test depending on normality and equal variance was used to check for statistical differences.

## Results

### Animals and Measurements

Fifteen of the 17 animals were analyzed. Two animals of the CCI group died during the experiment, one from pneumothorax and one related to CCI. Eight control and seven CCI animals were used for probe evaluation. Given that four MPBS were implanted per pig and the protocol consisted of five challenges, 20 recorded challenges were obtained per animal.

The different modules were checked for predefined inappropriate reactivity or implausible values. This was classified as device malfunctioning, and respective challenges were excluded. Moreover, artificially altered measurements by manipulation of the setup (e.g., accidental probe movement) during the protocol were excluded. 20.2% (ptiO_2_), 15.1% (ICP), and 12.8% (CBF) of measurements were affected.

The Hemedex probe exhibited multiple periods of measurement interruption per experiment. These were more frequent the further the protocol had progressed. In total, 21.4% of single measurement points distributed over 50 different challenges (68.5% of all challenges) were affected. Figure 7 (supplementary material) demonstrates exemplarily CBF values measured by MPBS and Hemedex in an individual animal of the control group.

No difficulties were experienced with MPBS probe implantation. Handling was equivalent to the similar-sized Neurovent-P probe. No major bleeding was macroscopically observed in cerebral cross sections after the experiment.

### In Vitro Measurements

The mean (± SEM) in vitro measurements of the ptiO_2_ sensors were 151.7 ± 4.3 mm Hg (MPBS) and 149.0 ± 1.8 mm Hg (Licox) in oxygen-enriched solution after equilibration. There was a statistically significant difference between the measurements in the oxygen-deprived solution (0.5 ± 0.1 mm Hg (MPBS), 1.2 ± 0.3 mm Hg (Licox), *p* = 0.031).

### Bland–Altman Plots

**Fig. 4:**
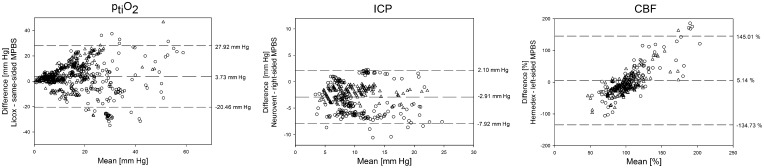
Bland–Altman plot. The Bland–Altman plots demonstrate the measurement difference between the reference probe and the MPBS on the *y*-axis (reference probe − MPBS) and the mean values of both on the *x*-axis [(reference probe + MPBS)/2]. Three dashed horizontal lines show the mean difference and 95% limits of agreement (mean difference ± 1.96 × SD). The control group is represented by a circle, the CCI group by a triangle

As shown in the Bland–Altman plots (Fig. 4), subtraction of MPBS from reference values revealed a mean difference of 3.7 mm Hg, −2.9 mm Hg, and 5.1% for ptiO_2_, ICP, and relative CBF, respectively. Accordingly, MPBS measured higher ICP but lower ptiO_2_ and CBF values than the reference probes.

### PtiO_2_ Key Challenges

**Fig. 5:**
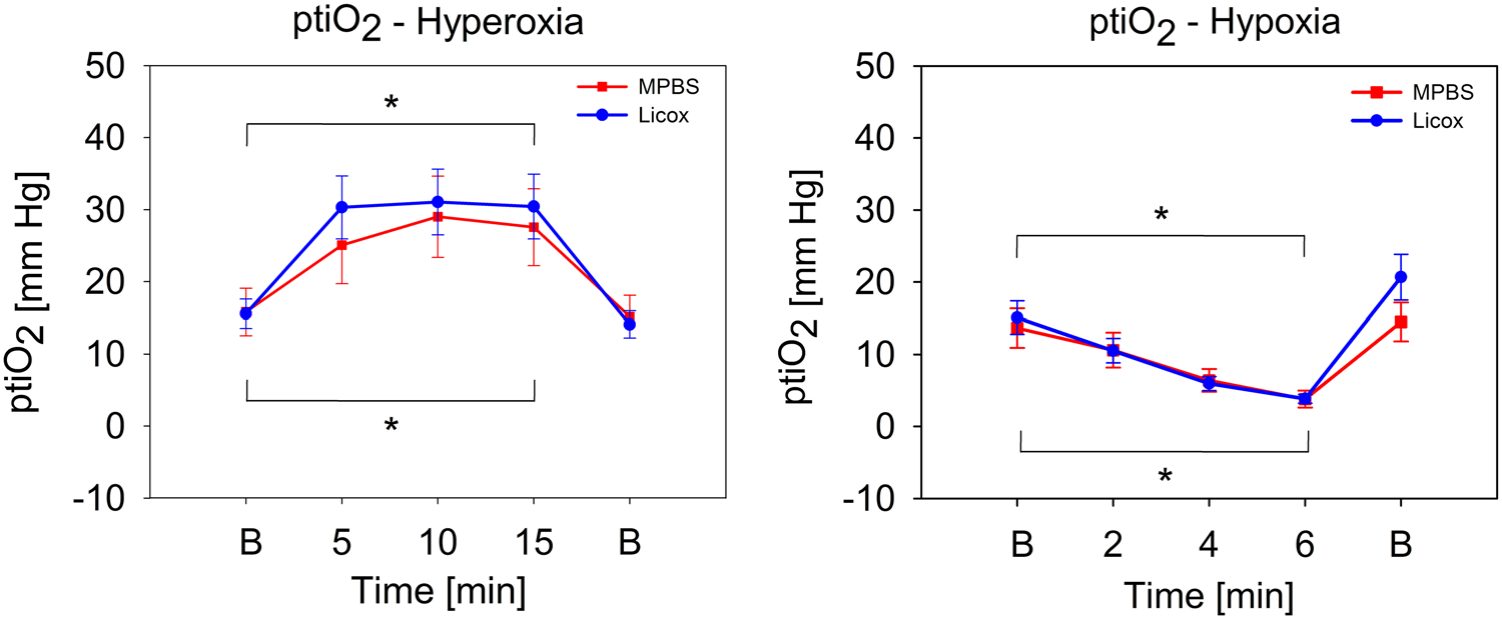
Key provocations for ptiO_2_ measurement evaluation. The line plots show mean values (± SEM) of all MPBS and Licox probes during key ptiO_2_ provocations for control animals. Asterisks (*) mark significant differences between the baseline and endpoint values (*p* < 0.05). There was no significant difference between the respective means of MPBS and Licox. B, baseline

Figure 5 shows mean values (± SEM) of MPBS in comparison with Licox probes in key challenges for ptiO_2_ in the control group. Both ptiO_2_ modules demonstrated a significant rise in ptiO_2_ from 15.8 ± 3.3 mm Hg (MPBS) and 15.6 ± 2.0 mm Hg (Licox) to 27.6 ± 5.3 mm Hg (MPBS; 74.7% increase, *p* = 0.041) and 30.4 ± 4.5 mm Hg (Licox; 94.9% increase, *p* = 0.011) after 15 min of hyperoxia. There was a mean increase in arterial partial pressure of oxygen (PaO_2_) from 147.6 ± 3.1 to 527.8 ± 35.5 mm Hg. Hypoxia led to a decrease in PaO_2_ from 130.7 ± 25.6 to 23.1 ± 10.6 mm Hg. The values measured by MPBS and Licox were 13.6 ± 2.7 and 15.1 ± 2.3 mm Hg at baseline, 3.8 ± 1.2 mm Hg (72.1% decrease, *p* < 0.001) and 3.8 ± 0.6 mm Hg (74.8% decrease, *p* < 0.001) after 6 min, respectively. This demonstrates a significant decline in ptiO_2_.

### ICP Key Challenges

**Fig. 6:**
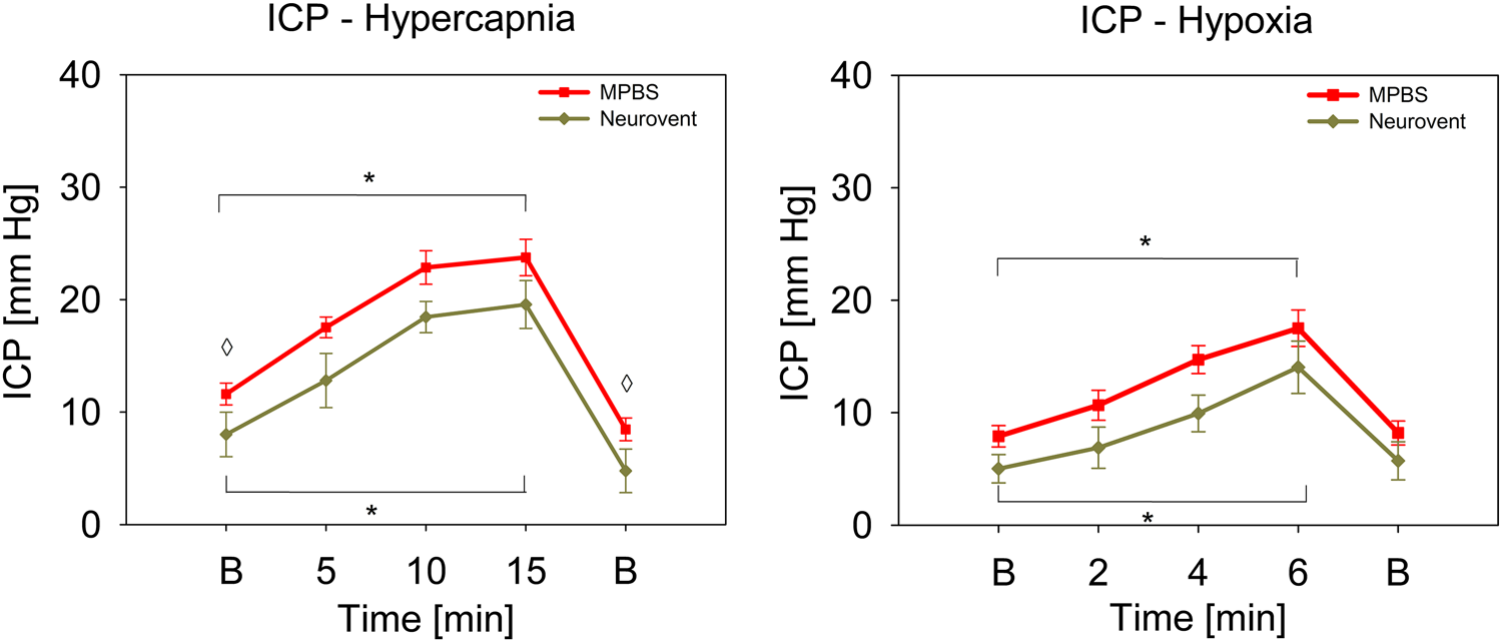
Key provocations for ICP measurement evaluation. The line plots show mean values (± SEM) of all MPBS and the Neurovent probe during key ICP provocations for control animals. Asterisks (*) mark significant differences between the baseline and endpoint values and diamonds (◊) between the two devices (*p* < 0.05). B, baseline

Figure 6 demonstrates the mean ICP values (± SEM) of MPBS and Neurovent for key ICP provocations in the control group. Hypercapnia resulted in a rise in arterial partial pressure of carbon dioxide (PaCO_2_) from 43.1 ± 1.7 to 103.0 ± 2.8 mm Hg after 15 min. ICP reacted with a significant increase from 11.6 ± 1.0 mm Hg (MPBS) and 8.0 ± 2.0 mm Hg (Neurovent) to 23.7 ± 1.6 mm Hg (MPBS; 104.3% increase, *p* < 0.001) and 19.6 ± 2.1 mm Hg (Neurovent; 145.0% increase, *p* = 0.004). Hypoxia also led to a significant increase in ICP from 7.9 ± 0.9 mm Hg (MPBS) and 5.0 ± 1.3 mm Hg (Neurovent) to 17.5 ± 1.6 mm Hg (MPBS; 121.5% increase, *p* < 0.001) and 14.0 ± 2.3 mm Hg (Neurovent; 180.0% increase, *p* = 0.004) after 6 min.

### PtiO_2_ Agreement Matrix

A dichotomized agreement matrix including a total of 890 ptiO_2_ measurement points of both control and CCI group revealed an observed agreement between MPBS and Licox of 0.63 and 0.68 applying thresholds of 15 or 20 mm Hg, respectively (Table 1, supplementary material). Deviating results were mainly caused by MPBS indicating a value below and Licox a value above the threshold. The other way around occurred only in 5.8 and 7.2% of cases applying thresholds of 15 or 20 mm Hg, respectively.

### Effect of CCI on CBF During Pharmacological Challenges

In relation to the initial baseline of the protocol, relative CBF as mean ± SEM of the CCI group was 98.8 ± 3.6% (MPBS) and 89.6 ± 7.4% (Hemedex) before dobutamine application. After 15 min of 15 µg/kg/h dobutamine application, relative CBF was 105.3 ± 6.9% (MPBS) and 104.7 ± 8.3% (Hemedex), which equals a nonsignificant rise (MPBS: *p* = 0.482, Hemedex: *p* = 0.198).

CBF values were 104.0 ± 7.1 and 80.4 ± 10.7% before norepinephrine challenge and increased to 113.5 ± 9.2 and 87.2 ± 9.6% under 15 min of 0.6 µg/kg/h norepinephrine application for MPBS (*p* = 0.431) and Hemedex (*p* = 0.501), respectively.

### Temporal Changes in Measurement

Figure 8 (supplementary material) demonstrates the mean (± SEM) of the differences between the values of the initial baseline and the baseline before hypoxia for different sensors of MPBS and respective reference probes in the control group. The difference was 2.5 ± 2.5 mm Hg (MPBS) and 0.5 ± 2.2 mm Hg (Licox) for ptiO_2_, 0.5 ± 0.8 (MPBS) and 1.1 ± 1.1 mm Hg (Raumedic) for ICP, and − 15.5 ± 12.7% (MPBS) and 23.2 ± 20.1% (Hemedex) for CBF measurement. There was no statistically significant difference between respective MPBS and reference probe deviations.

## Discussion

### The Relevance of Multiparametric Intraparenchymal Neuromonitoring

Several studies were able to show a reduced mortality linked to sole ICP monitoring [2–5]. However, other trials reported unchanged or even elevated mortality as well as prolonged duration of mechanical ventilation when ICP monitoring and guided therapy were applied [17–19]. Such conflicting results might be due to cerebral hypoxia despite an apparently adequate CPP [20]. By adding ptiO_2_ measurement to ICP monitoring and keeping ptiO_2_ values above 25 mm Hg, mortality could be reduced [21]. Some studies confirmed reduced mortality and morbidity in relation to a ptiO_2_-guided treatment [6–9]. However, missing beneficial effects on the outcome have been published as well [22, 23].

Simultaneous monitoring of ICP and ptiO_2_ with a single catheter is a substantial progress. This prevents additional complications and effort, which go along with implantation of several different probes. Combining this lineup with direct CBF measurement, e.g., LDF, can be a useful addendum for ischemia detection [24, 25]. Monitoring the described parameters simultaneously draws a more complete picture of the pathological changes taking place in brain-injured patients and can lead to better adjusted therapeutical strategies.

### A Methodical Approach to the Standardized Evaluation of Multiparameter Probes

The evaluation of a new measurement device is based on comparison with an already established technology. In case of multiparameter brain sensors, different reference probes are necessary for the respective parameters. For translational purposes, proper function of tested sensors should ideally be evaluated in vivo during standardized physiological challenges under both physiological and pathologic conditions.

Licox and Neurovent-P are well-established probes considered as ‘gold standard’ and adequate references since these devices have been well known by clinicians for years [26, 27]. Therefore, we preferred these probes over the relatively new Neurovent-PTO, which is also a multiparametric device for ICP and ptiO_2_ [10]. The already published evaluations of Neurovent-PTO also used Licox probes as Ref. [10, 11, 28]. Location of probes was mainly influenced by the location of the craniectomy. Ipsilateral probes were positioned as close as possible to the traumatic area with enough space between them to minimize interference. Therefore, Neurovent-P probe was implanted on the contralateral side since ICP is a more systemic parameter. The two additional MPBS and second Licox probe were inserted contralateral to allow for hemispheric-specific analysis.

CCI was performed with the intention to induce an additional factor of heterogeneity between different animals including alterations in cerebrovascular autoregulation. The validity of the study should have been increased by a broader range of values being available for Bland–Altman analysis. Moreover, the translational value has been enhanced with CCI as a model for a clinically relevant condition.

Two pharmacological challenges were included in the protocol mainly for CBF manipulation. Mean arterial pressure (MAP) dipped under dobutamine, whereas stable to slightly increased MAP was observed during norepinephrine application. An increase in cardiac index (CI) and heart rate was induced by both drugs. However, CCI animals did not show the expected major changes in CBF. Possibly, CCI was not severe enough to impair arterial autoregulation sufficiently or probe location was too distant.

Key provocations for ptiO_2_ were hyperoxia and hypoxia. As expected, alterations of PaO_2_ led to a significant increase or decrease in ptiO_2_. This was detected by both MPBS and Licox. ICP was mainly influenced by hypercapnia and hypoxia. Increased PaCO_2_ as well as decreased PaO_2_ acted as vasodilative agents leading to a significant rise in ICP which was also noticed by both monitoring devices.

Generally, methodical principles and considerations which were described before are now confirmed with an increased number of animals and measurements [13]. The animal model and protocol generally provide a feasible basis for future testing of neuromonitoring devices.

### Handling of the MPBS

The specification of the MPBS dimension is comparable to the well-established Neurovent-P probe, and insertion procedure was similar. The recently clinically induced multiparametric device Neurovent-PTO is stated with the same dimension. Since the non-flexible steel shaft is equally in length to the planned insertion depth in the utilized animal model, this was no hindering factor. In clinical application, a bolt device would seem to be feasible. No clinically relevant complications like intraparenchymal hemorrhage associated with MPBS insertion were registered.

### Evaluation of the ptiO_2_ Sensor

The ptiO_2_ and temperature sensors were compared with the well-established Licox probe (Integra Neuroscience) [27]. In our experiments, average in vivo ptiO_2_ values measured by Licox were 3.73 mm Hg higher than MPBS values. Certainly, attention should be paid to relatively broad limits of agreement. In comparison, measurement differences of other multiparameter probes to reference sensors seem to be similar:

The Neurovent-PTO monitor (Raumedic) combines the measurement of ICP, ptiO_2_—also by oxygen quenching—and temperature. In the literature, evaluation of this catheter showed higher mean ptiO_2_ values compared to Licox [10, 11, 28]. Differences were 6.3 mm Hg in a porcine model, 1.24 mm Hg (CI −25.1 to 22.6 mm Hg) in intensive care patients, and 6.1 mm Hg (CI −32.1 to 20.0 mm Hg) in FiO_2_- and MAP-challenged patients. An unequal sampling area size (Licox: 13 mm^2^; Neurovent-PTO: 22 mm^2^) and the oxygen consumption of the Clark electrode of the Licox probe were provided as possible explanations for higher Neurovent values. However, in our experiments, MPBS values were lower than Licox values even though MPBS and Neurovent-PTO have a similar technology, and the sampling size of the MPBS (13 mm^2^) and Licox is the same. Possibly, the oxygen consumption of the Clark electrode has less influence than expected and oxygen quenching generally measures lower values, which has been disguised in the Neurovent-PTO due to the larger sampling area.

The Paratrend/Neurotrend probe is another multiparameter catheter consisting of a fluorescent pO_2_ as well as ptiCO_2_, pH, and temperature sensors (Diametrics Medical Inc./Codman&Shurtleff) [29, 30]. This probe has been tested in brain-injured patients and showed higher values than the Licox sensor with mean differences of < 5 mm Hg [29].

Low ptiO_2_ values are a crucial indicator for cerebral hypoxia. Longer periods of a ptiO_2_ at or below 15 mm Hg were shown to increase the likelihood of death in ICU patients [31]. The agreement between MPBS and Licox in this critical ptiO_2_ range appears more satisfactory than in higher ptiO_2_ levels. The mean measurement difference in a range below 20 mm Hg was 4.0 mm Hg with almost halved limits of agreement (− 9.6 to 17.7 mm Hg). The most deviating values were recorded during hyperoxygenation. Analogously, in vitro testing showed a wider distribution of MPBS values in oxygen-enriched solution. Low oxygen levels were measured with only a small variance (0.5 ± 0.1 mm Hg). The increased sensitivity of MPBS in this lower range is attributable to the fact that fluorescence lifetime is longest at low ptiO_2_. A direct comparison of MPBS with another oxygen quenching probe might be worthwhile.

The clinical relevance of these deviations is depicted by a dichotomized agreement matrix showing an agreement of 0.63 and 0.68 for thresholds of 15 or 20 mm Hg, respectively. Given that deviating results were mainly caused by MPBS indicating a value below and Licox a value above the threshold, ptiO_2_ measurement by the oxygen quenching module of MPBS might be interpreted as more conservative. Clinically, this could lead to a possible overtreatment in comparison with Licox-guided treatment as the current standard probe. On the contrary, applying, e.g., a threshold of 15 mm Hg, a Licox-indicated treatment would be ‘missed’ by MPBS only in 5.8% of measurements. Further studies seem to be useful to clarify whether oxygen quenching technology might be a more sensitive technology for cerebral ischemia detection.

The temporal measurement deviation—evaluated at the baseline after hypercapnia—was higher in MPBS than in Licox. However, it is debatable whether this is attributable to a device-related drift. PtiO_2_ as a relatively local parameter is susceptible to environmental changes, and there might still have been an influence of the previous challenge due to disturbances in blood gases and vascular tone. Moreover, changes in the experimental setup like, e.g., brain temperature, could have affected the measurement.

The ptiO_2_ sensor of the MPBS exhibited improper function in 20.2% of applications. This exceeds the reported Licox error rate of 13.6% [32]. The error rate of the Neurovent-PTO ptiO_2_-module was reported to be 40% but was surveyed in intensive care patients and included handling errors [10]. However, Licox error rate was only 6.7% in the same setup, and accordingly, the single probe showed considerably less handling errors [10]. Dropouts in our experiments were possibly due to impairment of the catheter during implantation. Another explanation for malfunctioning could be clot creation around the tip. Such values and values linked to artificially caused measurement alteration by manipulation at the surgical site were excluded.

### Evaluation of the ICP Sensor

The ICP values of the MPBS were higher than the values of the Neurovent-P probe (Raumedic) with an average difference of 2.9 mm Hg. This deviation might be partially due to methodical issues like minor calibration errors. Moreover, the different probe locations with varying physiological or surgical conditions probably also have led to divergent measurements. As displayed in the key challenges (Fig. 6), the bias was mainly based on divergent baselines, whereas the extent of reaction after provocations was quite similar. However, the limits of agreement of − 7.9 to 2.1 mm Hg may imply a potential lack of both accuracy and precision.

With regard to other multiparameter probes, a comparison between the Neurovent-PTO catheter and an ICP reference probe does not exist. The ICP sensor technology is basically consistent with the Neurovent-P probe, which is already an established monitor [26]. However, confirmation of functionality also in the multiparameter setting, which demands technical alterations, would have been of interest.

The error rate of the ICP sensor was higher in the MPBS system (15.1%) than reported in the Neurovent-PTO probe (10%) [10]. Similar to the ptiO_2_ module, implantation-related impairments are possible. Values collected during methodical errors due to manipulation were excluded from analysis. Moreover, sufficient calibration of some probes was not achieved in some early animals due to an improperly functioning calibration box which was replaced later. The temporal measurement deviation of MPBS was rather small.

### Evaluation of the CBF Sensor

The comparison between the CBF sensors is based on relative flow changes since LDF cannot account for blood flow measurements in absolute terms. The change in CBF was set in relation to the baseline readings before each challenge. Altogether, the Hemedex reference probe measured 5.14% greater changes in blood flow. The linear distribution in the CBF Bland–Altman plot (Fig. 4) demonstrates a systemic bias between the two probes. Differences proportionally increase with the extent of relative change.

An advantage of intraparenchymal monitoring is that small changes can be well detected in real time. However, only a specific local area undergoes analysis. LDF can observe an area of about 1 mm^3^ [33]. Consequently, proximity to major vessels or implantation-associated bleeding can influence the measured CBF enormously. Therefore, varying probe insertion locations and implantation depth influence the evaluation, which attributes to the broad limits of agreement.

The deviation between the initial and late baseline was considerably high but might be rather attributed to, for example, microenvironmental changes than to a major drift, particularly given that it was present in both probes with different underlying measurement methods. From a technical point of view—at least for LDF—it should be a method with no substantial device-related drift, given that there is no membrane, electrode, or fluorophore suffering from attrition. A physiological basis for temporal changes, e.g., CBF tends to slowly increase in the aftermath of probe insertion as the microcirculation recovers from the physical insult, appears more likely. Equally, CBF can be influenced by brain temperature. Particularly Hemedex is more likely to be directly affected by environmental temperature changes. LDF would be indirectly affected by temperature-induced changes in blood flow.

The CBF module of the MPBS exhibited an error rate of 12.8%, and hence, LDF can be considered as a relatively failure-resistant technology. Indeed, the main difficulties with CBF measurement were caused by the Hemedex reference probe utilizing thermal diffusion. The Hemedex probe stopped measuring for some minutes several times during the experiments. This might have been caused by an automatic recalibration process [14]. Moreover, measurement interruption may also have been due to elevated temperatures in the measurement areal and subsequent avoidance of overheating of the brain tissue by the system [34]. Retrospectively, we consider the Hemedex probe as a suboptimal reference for this study due to its lack of continuity.

### General Limitations of this Study

A main limitation is the spatial differences between different probes. This especially affects ptiO_2_ and CBF measurements since ICP is a more systemic parameter. Probe location might have been in different vascular territories, and particularly CBF is influenced by the microvascular environment as described above.

Another potential source of bias is implantation depth due to differences in ptiO_2_ between cortical gray matter and white matter. Standardized depth was 15 mm in the experiments. However, given the different probe designs, the distance from surface to ptiO_2_ sampling area has varied between the different probes. As depicted in Fig. 3, the ptiO_2_ sampling area of the MPBS begins directly after the LDF module, whereas the distance from tip to sensitive area is 5 mm in Licox. Possibly, there were also unregistered accidental variations in depth.

Additionally, the diameters of the probes were different. This may have led to more microtrauma or microbleeding affecting the measurement in MPBS probes in comparison with Licox or Hemedex, which had smaller diameters.

As described in detail above, CBF evaluation was limited due to discontinuous measurement of the reference probe. Moreover, CBF was insufficiently challenged by the pharmacological provocations.

This study represents only a limited time frame, resulting in two limitations. First, it is possible that time from implantation to first challenge was not long enough to allow for perfect equilibration. In order to minimize this bias, stabilization of temperature and ptiO_2_ was awaited before starting the protocol. Second, measurement quality after days and a possible long-term drift could not have been assessed with this study.

## Conclusions

The demonstrated in vivo data of the MPBS document the ability to measure ptiO_2_, CBF, and ICP in a single catheter. Even though the measured values exhibited a certain deviation in comparison with reference probes, the performance was mostly analogous to other multiparameter devices. However, further evaluation and potential improvement of the device are necessary before it can reliably be used for clinical decision making.

Overall, the MPBS is a promising technology. Potentially, it could be a future gain for the management of brain-injured patients by enabling multiparameter neuromonitoring and allowing for new adapted therapy modalities. The suitability of the animal model and protocol for multiparameter probe evaluation were confirmed.

## Electronic supplementary material

Below is the link to the electronic supplementary material.

Supplementary material 1 (DOCX 13 kb)

Figure 7 Continuous CBF measurement in an individual animal. The graph shows CBF values measured by MPBS and Hemedex in an individual animal of the control group. Discontinuous measurement of Hemedex can be observed (TIF 1443 kb)

Figure 8 Temporal measurement deviation. The bar chart shows the mean (±SEM) difference between the initial baseline and the baseline between hypercapnia and hypoxia for the different MPBS modules and reference probes. There was no significant difference between the means of MPBS and respective reference probes (*p* > 0.05) (TIF 1469 kb)

## Abbreviations

CBF: Cerebral blood flow
CCI: Controlled cortical impact
CI: Cardiac index
CPP: Cerebral perfusion pressure
ICP: Intracranial pressure
LDF: Laser Doppler flowmetry
MAP: Mean arterial pressure
MPBS: Multiparameter brain sensor
PaCO_2_: Arterial partial pressure of carbon dioxide
PaO_2_: Arterial partial pressure of oxygen
ptiO_2_: Brain tissue oxygenation
TBI: Traumatic brain injury
