# Supplementary material for: Evaluation of a New Multiparameter Brain Probe for Simultaneous Measurement of Brain Tissue Oxygenation, Cerebral Blood Flow, Intracranial Pressure, and Brain Temperature in a Porcine Model
Source: Neurocrit Care. 2018 Jun 11;29(2):291–301. doi: 10.1007/s12028-018-0541-9 (PMC6208836; doi:10.1007/s12028-018-0541-9)
Supplement: Supplementary file 1 — Supplementary material 1 (DOCX 13 kb) [file 12028_2018_541_MOESM1_ESM.docx]

Supplementary material, Table 1.

**PtiO_2_ agreement matrix for thresholds of 15 mm Hg and 20 mm Hg**

|  |  | **Licox** | **Licox** |  |
| --- | --- | --- | --- | --- |
|  |  | **>15** | **<15** |  |
| **MPBS** | **>15** | **220 (24.7%)** | **52 (5.8%)** | **272** |
| **MPBS** | **<15** | **276 (31.0%)** | **342 (38.4%)** | **618** |
|  |  | **496** | **394** | **890** |

|  |  | **Licox** | **Licox** |  |
| --- | --- | --- | --- | --- |
|  |  | **>20** | **<20** |  |
| **MPBS** | **>20** | **114 (12.8%)** | **64 (7.2%)** | **178** |
| **MPBS** | **<20** | **224 (25.2%)** | **488 (54.8%)** | **712** |
|  |  | **338** | **552** | **890** |
