# Supplementary figures and images for: Evaluation of a New Multiparameter Brain Probe for Simultaneous Measurement of Brain Tissue Oxygenation, Cerebral Blood Flow, Intracranial Pressure, and Brain Temperature in a Porcine Model
Source: Neurocrit Care. 2018 Jun 11;29(2):291–301. doi: 10.1007/s12028-018-0541-9 (PMC6208836; doi:10.1007/s12028-018-0541-9)

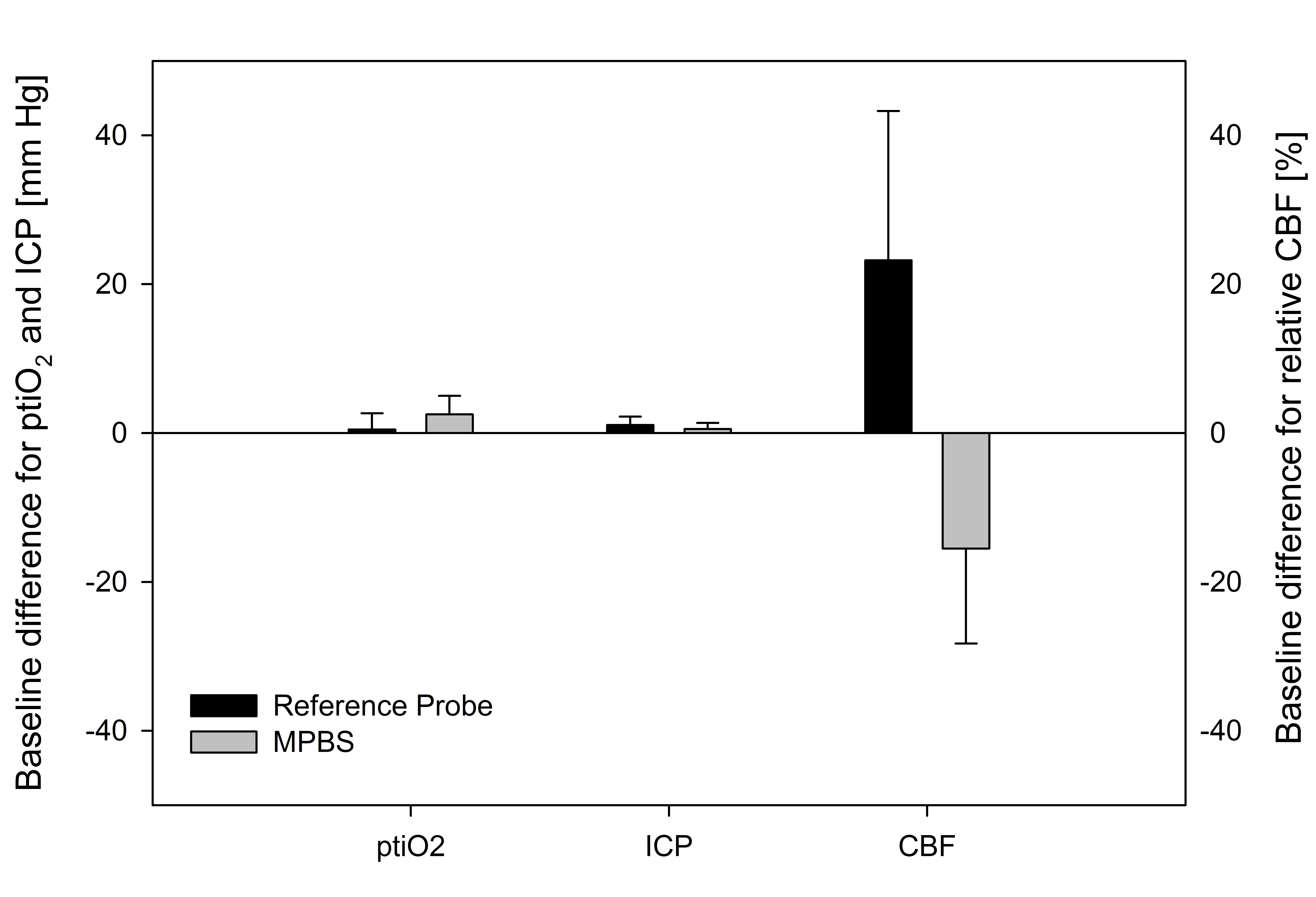

Supplement: Supplementary file 3 — Temporal measurement deviation. The bar chart shows the mean (±SEM) difference between the initial baseline and the baseline between hypercapnia and hypoxia for the different MPBS modules and reference probes. There was no significant difference between the means of MPBS and respective reference probes (p > 0.05) (TIF 1469 kb) [file 12028_2018_541_MOESM3_ESM.tif]
